# Plateau Hypoxia Influences Mouse Circadian Behavior by Remodeling the Hypothalamic Transcriptional Landscape

**DOI:** 10.3390/cells15151335

**Published:** 2026-07-25

**Authors:** Chang Lu, Jingjing Liu, Zeqi Li, Tingting Wang, Chengpu Wang, Jiaqi Gao, Zhaohong Nie, Yawen Yin, Bo Cui, Bo Fu

**Affiliations:** 1 Graduate School, Tianjin University of Traditional Chinese Medicine, Tianjin 301617, China; luchang@tjutcm.edu.cn (C.L.);; 2 Academy of Military Medical Sciences, Tianjin 300050, China; 3 College of Biological Sciences, China Agricultural University, Beijing 100193, China

**Keywords:** hypoxia, circadian rhythm, hypothalamus, transcriptome, relaxin signaling

## Abstract

**Highlights:**

**What are the main findings?**

- High-altitude exposure transiently reduced locomotor activity and delayed behavioral circadian phase in mice without altering the free-running period.
- High-altitude exposure extensively remodeled the rhythmic transcriptional landscape of the whole hypothalamus, with altered temporal expression of core clock genes and identification of relaxin-related signaling as a candidate pathway associated with hypoxia-induced transcriptional remodeling.

**What are the implications of the main findings?**

- The locomotor response to high-altitude exposure varies with circadian phase, with greater suppression observed during the active phase.
- Endogenous circadian timing influences behavioral responses to high-altitude exposure and provides a rationale for developing time-informed strategies for altitude exposure, acclimatization, and hypoxic training

**Abstract:**

Background: Hypoxia signaling and the circadian clock exhibit extensive molecular crosstalk, but how endogenous circadian timing influences behavioral responses during high-altitude exposure remains incompletely understood. Methods: Male C57BL/6J mice were exposed to hypobaric hypoxia equivalent to an altitude of 6000 m. Wheel-running activity was continuously monitored under 12 h light/12 h dark (LD) cycle and constant-dark conditions to evaluate locomotor activity, rhythm amplitude, circadian phase, and free-running period. After 48 h of exposure, whole-hypothalamus samples were collected across the circadian cycle for time-series RNA sequencing, rhythmicity and pathway-enrichment analyses, and qPCR validation. In a separate experiment, hypoxia was initiated at ZT2 or ZT14 to assess circadian-phase-dependent behavioral responses. Results: High-altitude exposure acutely reduced locomotor activity and rhythm amplitude and subsequently delayed behavioral circadian phase under a LD cycle, whereas the free-running period under constant darkness remained unchanged. Transcriptomic analysis revealed extensive remodeling of rhythmic gene expression in the whole hypothalamus, involving multiple signaling and metabolic pathways. Relaxin-related signaling emerged as a candidate pathway associated with hypoxia-induced temporal transcriptional remodeling, and selected core clock genes and relaxin pathway-associated genes showed altered temporal expression profiles. Furthermore, exposure initiated at ZT14 produced greater locomotor suppression than exposure initiated at ZT2. Conclusions: High-altitude exposure disrupts behavioral circadian organization and remodels hypothalamic rhythmic transcription. These findings suggest that endogenous circadian timing modulates behavioral sensitivity to hypoxic stress.

## 1. Introduction

Circadian rhythms are endogenous oscillations with a period of approximately 24 h that align behavior, physiology, and gene expression with recurring environmental cycles. In mammals, the circadian system regulates sleep/wake behavior, locomotor activity, hormone secretion, body temperature, feeding, and energy metabolism [1,2,3]. At the molecular level, circadian rhythms are generated by transcription–translation feedback loops (TTFL) in which the CLOCK/BMAL1 complex activates Period and Cryptochrome genes, whose protein products subsequently repress CLOCK/BMAL1-dependent transcription [2,3]. The mammalian circadian system is hierarchically organized. The suprachiasmatic nucleus (SCN) of the hypothalamus serves as the principal central pacemaker, receiving photic input from the retina and synchronizing peripheral oscillators through neural, endocrine, metabolic, and behavioral signals [4]. Circadian regulation within the hypothalamus, however, extends beyond the suprachiasmatic nucleus. Other hypothalamic regions contribute to the control of arousal, autonomic function, thermoregulation, feeding, energy expenditure, and neuroendocrine responses. The hypothalamus therefore represents a key interface between circadian timing and physiological adaptation to environmental stress [5,6].

Although light is the dominant entraining signal, nonphotic cues, including feeding, exercise, temperature, hormones, and oxygen availability, can also influence circadian timing [7,8,9]. Oxygen is particularly relevant because tissue oxygenation varies across the daily cycle in association with rhythmic changes in activity, blood flow, ventilation, and metabolism. Experimental studies further indicate that fluctuations in oxygen availability can act as timing signals and modulate molecular clocks through hypoxia-inducible factor-dependent mechanisms [7,8]. Conversely, core circadian components regulate hypoxia-responsive transcription and metabolic adaptation, demonstrating reciprocal crosstalk between the circadian clock and hypoxia signaling [10].

High-altitude exposure imposes a sustained reduction in oxygen availability and affects multiple physiological systems. It is commonly associated with reduced physical performance, fatigue, sleep disruption, altered thermoregulation, impaired cognition, and changes in energy metabolism, many of which are themselves under circadian control. Previous studies have shown that hypoxic responses vary with time of day and that sustained hypoxia can alter rhythmic gene expression in peripheral tissues and circulating cells [11,12,13,14]. Hypoxia has also been reported to alter melatonin timing and promote temporal misalignment among tissues, supporting the possibility that reduced oxygen availability can disrupt circadian organization at the whole-organism level [15]. It is unclear whether sustained hypobaric hypoxia primarily affects the phase, amplitude, or intrinsic period of behavioral circadian rhythms.

In the present study, we examined the effects of high-altitude exposure on locomotor circadian rhythms in mice and characterized associated time-dependent transcriptional changes in the whole hypothalamus. Wheel-running activity was monitored under light–dark and constant-dark conditions to distinguish changes in behavioral circadian phase from changes in the free-running period. Time-series RNA sequencing was used to identify hypoxia-associated alterations in rhythmic gene expression and pathway enrichment across the circadian cycle. We also initiated high-altitude exposure during either the inactive or active phase to determine whether the locomotor response varied with circadian phase. High-altitude exposure acutely suppressed locomotor activity, delayed behavioral circadian phase without significantly altering the free-running period, and broadly reorganized the rhythmic transcriptome of the whole hypothalamus. Greater locomotor suppression was observed when exposure began during the active phase, while relaxin-associated signaling emerged as a candidate pathway requiring further functional validation.

## 2. Materials and Methods

### 2.1. Animals

Male C57BL/6J mice aged 6–8 weeks and weighing 20–25 g were obtained from Vital River Laboratory Animal Technology Co., Ltd. (Beijing, China). Animals were housed under controlled environmental conditions at 22 ± 2 °C, and a 12 h light/12 h dark cycle, with lights on at zeitgeber time 0 (ZT0) and lights off at ZT12. Food and water were available ad libitum unless otherwise specified. Before experimentation, mice were allowed to acclimate to the housing environment for at least 7 days. Mice were randomly allocated to control and high-altitude exposure groups before the experiments. Randomization was performed using a simple random allocation procedure, and animals with comparable age and body weight were distributed among groups. All animal procedures were approved by the Institutional Animal Care and Use Committee of the Tianjin Institute of Environmental and Operational Medicine and were conducted in accordance with relevant institutional and national guidelines for the care and use of laboratory animals.

### 2.2. Plateau Hypoxia Environment Exposure

Plateau hypoxia environment exposure was performed by a multifunctional environmental simulation chamber (Guizhou Fenglei Aviation Ordnance Co., Ltd., Anshun, China) [16]. The chamber was decompressed to a barometric pressure corresponding to an altitude of 6000 m to carry out experiments. The normoxic control group was housed under otherwise comparable environmental conditions at local atmospheric pressure.

### 2.3. Wheel-Running Activity and Circadian Rhythm Analysis

Mice were individually housed in cages equipped with running wheels. Wheel-running activity was continuously recorded using clocklab system (Actimetrics, Wilmette, IL, USA) and collected in 5 min intervals [17]. Mice were first entrained under a 12 h light/12 h dark cycle for 7 days. After baseline recording, mice assigned to the high-altitude exposure group were transferred to the hypobaric chamber simulating 6000 m altitude, whereas control mice remained under normoxic conditions. Wheel-running activity was recorded continuously throughout baseline and exposure periods.

Double-plotted actograms, daily activity, circadian phase, and amplitude were generated using Clocklab analysis 6 software. The free-running period was calculated after mice were transferred to constant darkness. All procedures conducted during the dark phase were performed under dim red light.

### 2.4. Circadian-Phase-Dependent High-Altitude Exposure

To determine whether the locomotor response to high-altitude exposure depended on circadian phase, exposure was initiated either at ZT2, during the light and normally inactive phase, or at ZT14, during the dark and normally active phase [18].

### 2.5. Hypothalamus Collection

For transcriptomic analysis, mice were exposed to simulated 6000 m high-altitude conditions or normoxia for 48 h and euthanized at 4 h intervals across one 24 h cycle at ZT0, ZT6, ZT12, ZT18 and ZT24. Each treatment-by-time-point group contained 3 mice independent biological replicates. To minimize handling-related variation, tissue collection was performed rapidly and in a consistent order across time points. Dark-phase sampling was conducted under dim red light. Mice were deeply anesthetized using avertin and perfused with ice-cold saline to reduce blood contamination. Brains were removed immediately and placed on an ice-cold surface. The whole hypothalamus was dissected according to brain alts. Tissue samples were snap-frozen in liquid nitrogen and stored at −80 °C until RNA extraction.

### 2.6. RNA Extraction, Library Preparation, and Sequencing

RNA extraction, library preparation, and sequencing procedures were performed as described below. Total RNA was extracted from hypothalamic tissue using TRIzol Reagent (Thermo Fisher Scientific, Carlsbad, CA, USA) according to the manufacturer’s instructions. RNA concentration and purity were assessed using a NanoDrop ND-2000 spectrophotometer (Thermo Fisher Scientific, Carlsbad, CA, USA), based on the A260/A280 absorbance ratio. RNA integrity was evaluated using an Agilent 4150 Bioanalyzer (Agilent Technologies, Santa Clara, CA, USA). Only RNA samples that met the predefined quality criteria were used for library construction (A260/A280 = 1.8–2.1, RIN ≥ 7.0).

Paired-end sequencing libraries were prepared from 1 μg of total RNA using the ABclonal mRNA-seq Library Preparation Kit (ABclonal, Wuhan, China) according to the manufacturer’s protocol. Polyadenylated mRNA was enriched using oligo magnetic beads and fragmented in first-strand synthesis reaction buffer at elevated temperature. First-strand cDNA was synthesized from the fragmented mRNA using random hexamer primers and reverse transcriptase. Second-strand cDNA synthesis was subsequently performed using DNA polymerase I and RNase H.

The resulting double-stranded cDNA fragments were end-repaired, adapter-ligated, and amplified by PCR. Amplified products were purified using the AMPure XP system, and library concentration, fragment-size distribution, and overall quality were assessed using an Agilent 4150 Bioanalyzer. Qualified libraries were sequenced on an MGISEQ-T7 platform to generate 150 bp paired-end reads.

### 2.7. RNA-Seq Data Processing and Gene-Expression Quantification

The data generated from Illumina NovaSeq 6000 were used for bioinformatic analysis [19,20,21,22]. Raw sequencing reads in FASTQ format were processed using in-house Perl scripts. Adapter-containing reads were removed, together with reads for which bases with quality scores ≤ 25 accounted for more than 60% of the total read length. Reads containing more than 5% undetermined nucleotides were also excluded. The remaining high-quality reads were retained as clean reads for subsequent analyses.

Clean reads were aligned to the mouse reference genome [GRCm38/mm10] using HISAT2 (https://daehwankimlab.github.io/hisat2/) with strand-specific alignment parameters where applicable. Gene-level read counts were generated using featureCounts (https://subread.sourceforge.net/) based on the corresponding genome annotation file. Gene expression was quantified as fragments per kilobase of transcript per million mapped reads (FPKM), calculated from gene length and the number of reads mapped to each gene. Raw gene-level counts were used for statistical analyses requiring count-based input. Differential expression analysis was performed using the DESeq2, DEGs with | log2 FC | > 1 and Padj < 0.05 were considered to be significantly different expressed genes.

### 2.8. Rhythmicity Analysis

Rhythmic gene expression was analyzed separately in the normoxia and high-altitude exposure groups using the MetaCycle package in R [23,24]. Expression data collected at ZT0, ZT6, ZT12, ZT18, and ZT24 were included in the analysis. All biological replicates were retained as individual observations at their corresponding zeitgeber times and were not averaged before analysis. Rhythmicity was assessed using the JTK function, with the candidate period fixed at 24 h. Genes with a JTK_pvalue < 0.05 from MetaCycle were classified as rhythmic.

Genes meeting the rhythmicity criterion only under normoxia, only under high-altitude exposure, or under both conditions were designated normoxia-associated, high-altitude exposure-associated, or shared rhythmic genes, respectively. These categories were used for exploratory comparisons. Because statistical significance in one condition and nonsignificance in the other do not constitute a formal test of differential rhythmicity, these classifications were not interpreted as definitive evidence of rhythm loss or gain.

Kyoto Encyclopedia of Genes and Genomes (KEGG) pathway enrichment analysis was performed to identify biological pathways overrepresented among the rhythmic genes [25]. KEGG pathway enrichment analysis was performed using the clusterProfiler R package (4.14.3) [26]. Pathways with adjusted *p* value < 0.05 were considered significantly enriched. Rhythmic gene sets identified under each experimental condition were analyzed separately. *p* values were adjusted for multiple comparisons using the Benjamini–Hochberg method, and pathways with an adjusted *p* value of <0.05 were considered significantly enriched.

### 2.9. Quantitative Real-Time PCR

Whole-hypothalamus tissues were collected from mice exposed to normoxia or high-altitude conditions for 48 h at ZT0, ZT6, ZT12, ZT18, and ZT24. Each treatment-by-time-point group contained three independent biological replicates, with each biological replicate obtained from an individual mouse. Total RNA was extracted from hypothalamic tissues using TRIzol reagent (Thermo Fisher Scientific, Carlsbad, CA, USA) according to the manufacturer’s instructions. RNA concentration and purity were assessed using a NanoDrop ND-2000 spectrophotometer (Thermo Fisher Scientific, Carlsbad, CA, USA). Equal amounts of total RNA (500 ng) were reverse-transcribed into cDNA using the PrimeScript RT Reagent Kit (Takara Bio, Kusatsu, Japan) according to the manufacturer’s protocol. qPCR was performed using iTaq Universal SYBR Green Supermix (Bio-Rad, Hercules, CA, USA) on an Applied Biosystems QuantStudio 5 Real-Time PCR System (Thermo Fisher Scientific, Waltham, MA, USA). Each biological sample was analyzed in duplicate technical reactions. Melt-curve analysis was performed after amplification to verify the specificity of the PCR products. Relative mRNA expression levels were calculated using the 2^−ΔΔCt method and normalized to *Actin* expression. Primer sequences are provided in Table 1.

### 2.10. Statistical Analysis

All data are presented as the mean ± standard error of the mean unless otherwise stated. Individual mice were treated as the biological experimental units. When the sample data met the criteria for a normal distribution and an equal variance, unpaired *t* tests were used to compare the two treatment groups; one-way ANOVA with Tukey’s multiple-comparisons test and two-way ANOVA with Dunnett’s multiple-comparisons test were used for multiple group comparisons. Otherwise, nonparametric tests were used. Differences with *p* values less than or equal to 0.05 were considered statistically significant. GraphPad PRISM 8.0 software was used for the statistical analysis.

## 3. Results

### 3.1. High-Altitude Exposure Alters Locomotor Circadian Rhythms in Mice

**Figure 1:**
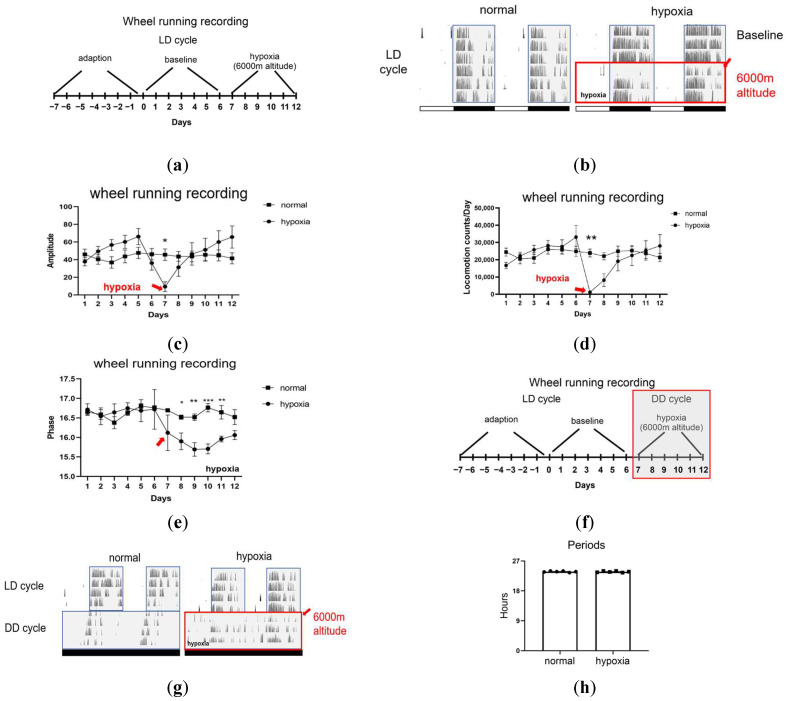
High-altitude exposure alters locomotor circadian rhythms in mice. (**a**) Experimental design for wheel-running recording under a LD cycle. Mice underwent 6 days of baseline recording under normoxia, followed by 6 days of hypobaric hypoxia equivalent to an altitude of 6000 m. (**b**) Representative double-plotted actograms recorded during baseline normoxia and high-altitude exposure. Black marks indicate wheel-running activity, gray shading denotes the dark phase, and the red box marks the period of high-altitude exposure. (**c**) Circadian rhythm amplitude during baseline and high-altitude exposure. The red arrow indicates the onset of hypoxia exposure on day 7. (**d**) Total daily locomotor activity during baseline and exposure. The *x*-axis indicates experimental day, and the *y*-axis indicates total wheel-running counts per 24 h. The red arrow indicates the onset of hypoxia exposure on day 7. (**e**) Changes in the phase of locomotor activity following the onset of high-altitude exposure. The red arrow indicates the onset of hypoxia exposure on day 7. (**f**) Experimental design for assessment of free-running rhythms under constant darkness. (**g**) Representative actograms recorded under LD cycle and constant-dark conditions. Black marks indicate wheel-running activity, gray shading denotes the dark phase during the LD cycle, and the red box marks the period of high-altitude exposure. (**h**) Free-running circadian period in DD cycle under normoxic and high-altitude exposure conditions. Data are presented as mean ± SEM; *n* = 6 mice per group. Comparisons between two groups were performed using an unpaired two-tailed *t* test, whereas time-course data were analyzed using two-way ANOVA followed by Dunnett’s multiple-comparisons test. * *p* < 0.05; ** *p* < 0.01; *** *p* < 0.001.

To characterize the effects of high-altitude exposure on circadian behavior, mice were individually housed in running-wheel cages and monitored under a LD cycle. Following a 6-day baseline recording period under normoxic conditions, mice were exposed for 6 days to a hypobaric environment equivalent to an altitude of 6000 m, while wheel-running activity was continuously recorded (Figure 1a,b). High-altitude exposure caused a pronounced reduction in circadian rhythm amplitude and total locomotor activity on the first day of exposure (Figure 1c,d). Both measures subsequently showed gradual recovery from the second day onward, although locomotor activity remained altered during the exposure period. In addition to the acute suppression of locomotor output, the phase of wheel-running activity was delayed beginning on the second day after exposure onset, and this delay persisted for at least three consecutive days under the LD cycle (Figure 1e).

To determine whether high-altitude exposure also altered the intrinsic period of the circadian oscillator, free-running rhythms were assessed under constant darkness (DD) following baseline entrainment (Figure 1f,g). No significant difference in free-running period was detected between the normoxic and high-altitude exposure groups (Figure 1h). Together, these findings indicate that high-altitude exposure transiently suppresses locomotor activity and rhythm amplitude and delays the phase of behavioral rhythms without detectably altering the endogenous free-running period.

### 3.2. High-Altitude Exposure Remodels Rhythmic Gene Expression in the Whole Hypothalamus

**Figure 2:**
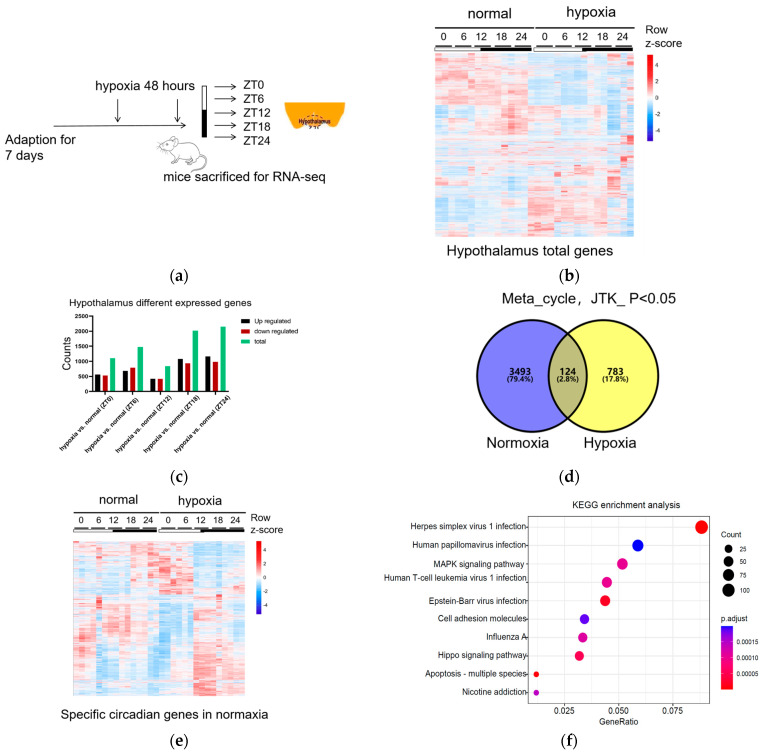

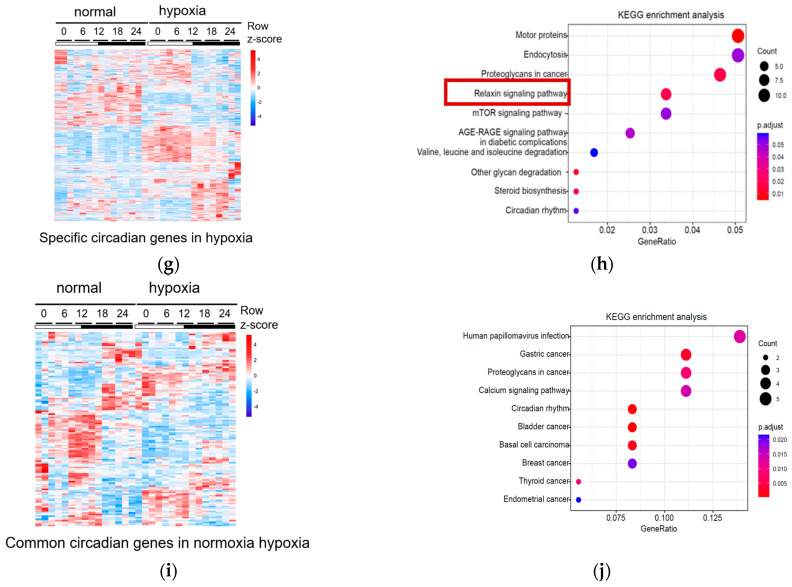
High-altitude exposure remodels the rhythmic transcriptional landscape of the whole hypothalamus. (**a**) Experimental design for time-series transcriptomic analysis. Whole-hypothalamus samples were collected after 48 h of normoxic or high-altitude exposure at ZT0, ZT6, ZT12, ZT18, and ZT24 (*n* = 3 mice each time/group). (**b**) Heatmap showing the temporal expression patterns of 21,349 detected genes under normoxic and high-altitude exposure conditions. Expression values were standardized by gene, with red and blue indicating relatively high and low expression, respectively. (**c**) Numbers of differentially expressed genes at each zeitgeber time point. Black, red, and green bars indicate upregulated, downregulated, and total differentially expressed genes, respectively. (**d**) Venn diagram showing the numbers and overlap of rhythmic genes identified under normoxic and high-altitude exposure conditions using MetaCycle with the candidate period fixed at 24 h. (**e**) Heatmap of genes meeting the rhythmicity criterion only under normoxia. (**f**) KEGG pathway enrichment analysis of genes meeting the rhythmicity criterion only under normoxia. The *x*-axis represents the GeneRatio, dot size represents the number of genes assigned to each pathway, and dot color represents the adjusted *p* value. (**g**) Heatmap of genes meeting the rhythmicity criterion only under high-altitude exposure. (**h**) KEGG pathway enrichment analysis of genes meeting the rhythmicity criterion only under high-altitude exposure. The *x*-axis represents the GeneRatio, dot size represents gene count, and dot color represents the adjusted *p* value. The red box highlights the relaxin signaling pathway, the key candidate pathway identified in this study. (**i**) Heatmap of genes meeting the rhythmicity criterion under both conditions. (**j**) KEGG pathway enrichment analysis of the shared rhythmic genes. The *x*-axis represents GeneRatio, dot size represents gene count, and dot color represents the adjusted *p* value.

To investigate the transcriptional changes associated with the behavioral circadian phase delay, mice were exposed to hypoxia for 48 h and whole-hypothalamus samples were collected every 6 h across a 24 h cycle at ZT0, ZT6, ZT12, ZT18 and ZT24 (Figure 2a). Transcriptomic analysis identified 21,349 expressed genes across the dataset (Figure 2b). The number of upregulated, downregulated and total differentially expressed genes varied across zeitgeber times, indicating that the hypothalamic transcriptional response to hypoxia was time-dependent (Figure 2c). Rhythmic transcripts were identified separately in the normoxia and high-altitude exposure groups using MetaCycle with a fixed 24 h period. Genes with a JTK_pvalue < 0.05 were classified as rhythmic. A total of 3493 genes met the rhythmicity criterion only under normoxia, 783 met the criterion only under hypoxia, and 124 were classified as rhythmic under both conditions (Figure 2d,e,g,i).

Genes meeting the rhythmicity criterion only under normoxia were enriched in KEGG pathways related to MAPK signaling, cell adhesion molecules, Hippo signaling, apoptosis, and nicotine addiction (Figure 2e,f). Genes classified as rhythmic only under high-altitude exposure were enriched in pathways associated with motor proteins, endocytosis, relaxin signaling, mTOR signaling, and circadian rhythm (Figure 2g,h). The 124 genes meeting the rhythmicity criterion under both conditions were enriched in circadian rhythm, calcium signaling, and several disease-associated pathways (Figure 2i,j). These results indicate broad condition-associated differences in rhythmic gene expression in the whole hypothalamus and identify several candidate pathways, including relaxin-related signaling.

### 3.3. High-Altitude Exposure Alters the Temporal Expression of Core Clock and Relaxin Pathway-Associated Genes

**Figure 3:**
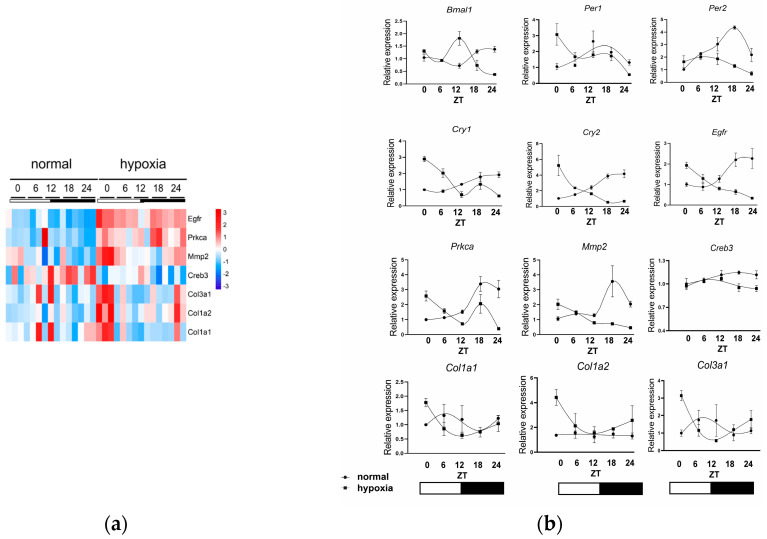
High-altitude exposure alters the temporal expression profiles of core clock genes and relaxin pathway-associated genes in the whole hypothalamus. (**a**) Heatmap showing the temporal expression patterns of selected relaxin pathway-associated genes under normoxic and high-altitude exposure conditions. (**b**) Quantitative real-time PCR analysis of core clock genes and selected relaxin pathway-associated genes at ZT0, ZT6, ZT12, ZT18, and ZT24 under normoxic and high-altitude exposure conditions. The data are shown as the mean ± SEM.

Among the pathways enriched in genes classified as rhythmic only under high-altitude exposure, relaxin signaling was selected for further exploratory analysis. Seven pathway-associated genes including *Egfr*, *Prkca*, *Mmp2*, *Creb3*, *Col1a1*, *Col1a2*, and *Col3a1* showed distinct temporal expression profiles between the normoxic and high-altitude exposure groups in the RNA-seq dataset (Figure 3a). qPCR analysis further revealed that high-altitude exposure altered the 24 h expression profiles of the core clock genes *Bmal1*, *Per1*, *Per2*, *Cry1*, and *Cry2*. Temporal expression patterns of the selected relaxin pathway-associated genes were also modified, with *Egfr*, *Mmp2*, *Col1a2*, and *Col3a1* showing particularly pronounced differences between the two conditions (Figure 3b).

Together, these results indicate that high-altitude exposure is accompanied by coordinated changes in the temporal expression of core clock genes and selected relaxin pathway-associated genes in the whole hypothalamus. However, the present expression data support only an association and do not establish a causal role for relaxin signaling in the behavioral response to high-altitude exposure.

### 3.4. Circadian Timing Modulates the Locomotor Response to High-Altitude Exposure

**Figure 4:**
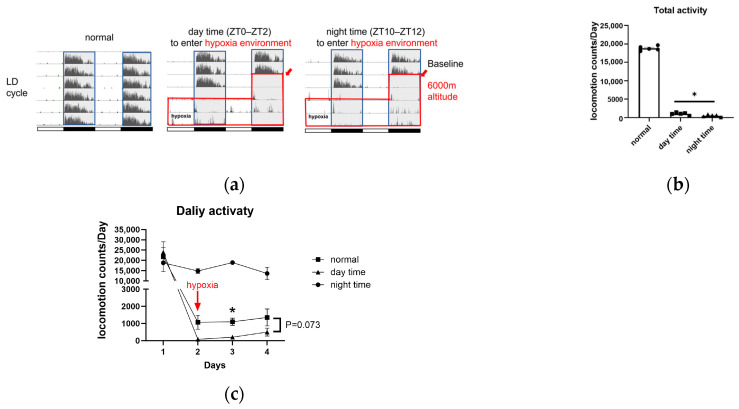
Locomotor responses to high-altitude exposure vary across circadian phases. (**a**) Representative double-plotted wheel-running actograms from mice maintained under normoxia or exposed to hypobaric hypoxia beginning at ZT2 or ZT14. ZT2 corresponds to the light and normally inactive phase, whereas ZT14 corresponds to the dark and normally active phase. Black marks indicate wheel-running activity, gray shading denotes the dark phase, and red boxes indicate the period of high-altitude exposure. (**b**) Total locomotor activity in the normoxia, ZT2-exposure, and ZT14-exposure groups during the defined observation period. The *y*-axis indicates wheel-running counts per day, and each point represents one mouse. (**c**) Daily locomotor activity before and after the onset of high-altitude exposure. The *x*-axis indicates experimental day, and the *y*-axis indicates wheel-running counts per day. The red arrow indicates the onset of hypoxia exposure. Squares, triangles, and circles represent the normoxia, ZT2-exposure, and ZT14-exposure groups, respectively. Data are presented as mean ± SEM; *n* = 5 mice per group. Panel (**b**) was analyzed using one-way ANOVA followed by Tukey’s multiple-comparisons test, and panel (**c**) was analyzed using two-way ANOVA followed by Dunnett’s multiple-comparisons test. * *p* < 0.05.

To determine whether the locomotor response to high-altitude exposure varied with circadian phase, hypobaric hypoxia was initiated at either ZT2, during the light and normally inactive phase, or ZT14, during the dark and normally active phase (Figure 4a). Representative actograms showed a marked reduction in wheel-running activity following the onset of high-altitude exposure in both groups (Figure 4a).

Total locomotor activity during the post-exposure observation period was lower in the ZT14-exposure group than in the ZT2-exposure group (Figure 4b). Analysis of daily activity further showed that wheel-running activity declined sharply after hypoxia onset in both exposure groups, with a greater reduction observed following exposure initiated at ZT14 (Figure 4c). These findings indicate that the behavioral response to high-altitude exposure varies according to circadian phase. Because ZT14 corresponds to the early active phase in nocturnal mice, the stronger reduction in wheel-running activity suggests that circadian state may modulate the behavioral impact of hypoxic stress.

## 4. Discussion

The present study investigated how high-altitude exposure influences locomotor circadian rhythms and time-dependent gene expression in the mouse hypothalamus. High-altitude exposure acutely reduced wheel-running activity and rhythm amplitude and was followed by a persistent delay in behavioral circadian phase under a LD cycle. In contrast, the free-running period under constant darkness was not significantly altered. Time-series transcriptomic analysis further revealed broad reorganization of rhythmic gene expression in the whole hypothalamus, including altered temporal profiles of core clock genes and genes associated with multiple signaling and metabolic pathways. In addition, locomotor responses differed when high-altitude exposure was initiated at ZT2 or ZT14. Together, these findings indicate that both behavioral and hypothalamic transcriptional responses to hypoxia vary with circadian phase.

A major finding was that high-altitude exposure delayed behavioral circadian phase without detectably altering the free-running period. Circadian phase and period represent distinct properties of the circadian system. The preservation of the free-running period suggests that the intrinsic pace of the circadian oscillator remained relatively stable under the conditions examined, whereas mechanisms involved in phase regulation, circadian output, or coupling among hypothalamic oscillators may have been more sensitive to hypoxia. This interpretation is consistent with previous evidence showing reciprocal interactions between hypoxia-inducible signaling and the molecular circadian clock [7,8,10,27,28]. However, wheel-running activity is a downstream behavioral output that can be strongly influenced by physiological state. Acute hypoxia may reduce exercise capacity, alter arousal, increase fatigue, and directly suppress movement. The delayed behavioral phase observed under the LD cycle may therefore reflect, at least in part, negative masking or reduced locomotor capacity rather than complete resetting of the central circadian pacemaker. The present data cannot distinguish among a shift in SCN phase, altered hypothalamic circadian output, disrupted coupling among oscillators, and direct behavioral suppression.

High-altitude exposure was also associated with extensive reorganization of rhythmic gene expression in the whole hypothalamus. Thousands of genes met the rhythmicity criterion only under normoxia, whereas a smaller group met the criterion only under high-altitude exposure. This pattern suggests redistribution of temporal regulation across biological pathways rather than uniform suppression of rhythmic transcription. Genes classified as rhythmic only under normoxia were enriched in pathways related to MAPK signaling, cell adhesion, Hippo signaling, and apoptosis, whereas genes classified as rhythmic only under high-altitude exposure were enriched in endocytosis, motor proteins, mTOR signaling, circadian rhythm, and relaxin signaling. These classifications should nevertheless be interpreted cautiously.

Relaxin signaling emerged as one of the candidate pathways associated with rhythmic expression under high-altitude exposure. Relaxin-family peptides and receptors are expressed in the mammalian brain and have been implicated in neuroendocrine regulation, stress responses, and neuromodulation [28,29]. Relaxin-related signaling also contributes to extracellular-matrix turnover and regulation of matrix metalloproteinase activity [30,31]. In this context, the altered temporal expression of *Mmp2*, *Col1a1*, *Col1a2*, and *Col3a1* raises the possibility that high-altitude exposure may reflect alterations in extracellular-matrix-related or neurovascular-associated transcriptional programs in the hypothalamus. Nevertheless, the present evidence is insufficient to establish that relaxin signaling mediates the behavioral phase delay. Relaxin-family peptides, their receptors, receptor activation, and downstream signaling activity were not directly measured. Relaxin signaling should therefore be regarded as a hypothesis-generating candidate rather than a confirmed mechanism.

The anatomical resolution of the transcriptomic analysis represents another important limitation. RNA-seq and qPCR were performed using whole-hypothalamus samples, which contain multiple anatomically and functionally distinct nuclei, as well as diverse neuronal, glial, vascular, and immune cell populations. The observed expression changes therefore cannot be assigned specifically to the SCN or to any other hypothalamic nucleus. Bulk expression patterns may also reflect divergent responses among hypothalamic regions or changes in cellular composition. In addition, changes in cell-type composition cannot be excluded in bulk transcriptomic analysis. This limitation is particularly relevant because the SCN primarily regulates circadian phase, whereas extra-SCN hypothalamic regions contribute to arousal, feeding, thermoregulation, autonomic function, and energy balance.

The difference in locomotor activity between exposure initiated at ZT2 and ZT14 further suggests that the behavioral response to high-altitude exposure varies with circadian phase. In nocturnal mice, ZT14 occurs early in the active phase, when spontaneous activity, oxygen consumption, sympathetic output, and metabolic demand are generally elevated. Exposure during this phase may therefore produce a greater mismatch between oxygen supply and physiological demand than exposure during the inactive phase. LD state, baseline activity, feeding history, sleep pressure, and prior energy expenditure all differ between ZT2 and ZT14. Absolute locomotor activity is also naturally higher during the dark phase, which may influence the apparent magnitude of suppression.

Several systemic effects of hypoxia may also contribute to the behavioral and transcriptional phenotypes. High-altitude exposure can alter core body temperature, food and water intake, body mass, energy expenditure, substrate utilization, sleep architecture, stress hormone secretion, ventilation, and cardiovascular function [32,33,34,35]. Each of these factors can independently influence wheel-running activity and circadian gene expression. For example, reductions in body temperature or food intake could alter both behavioral phase and hypothalamic transcription through temperature- and nutrient-sensitive pathways [9,36]. Changes in energy metabolism may also contribute to the enrichment of mTOR and related metabolic signaling pathways [37]. Because these physiological variables were not measured, they cannot be excluded as mediators or confounding factors. Future studies should incorporate telemetry-based body-temperature monitoring, automated food-intake measurements, body-mass tracking, indirect calorimetry, blood-gas analysis, oxygen-saturation monitoring, and assessment of sleep–wake state. Pair-feeding experiments would also help distinguish direct effects of hypoxia from secondary effects caused by reduced food intake.

In summary, high-altitude exposure altered behavioral circadian phase and broadly reorganized rhythmic gene expression in the whole hypothalamus without detectably changing the free-running period. The behavioral response also differed between exposure initiated at ZT2 and ZT14, suggesting that circadian phase influences the locomotor response to hypoxic stress. Relaxin-related signaling emerged as a candidate component of the transcriptional response, although its functional role remains to be established.

## 5. Conclusions

High-altitude exposure acutely reduced locomotor activity and rhythm amplitude and delayed behavioral circadian phase in mice without significantly altering the free-running period. These behavioral changes were accompanied by broad reorganization of rhythmic gene expression in the whole hypothalamus. Relaxin-related signaling emerged as one of several candidate pathways associated with the temporal transcriptional response. Locomotor responses also differed between exposure initiated at ZT2 and ZT14, indicating that the behavioral effects of high-altitude exposure vary with circadian phase. Together, these findings establish an association among high-altitude hypoxic stress, circadian phase, and hypothalamic rhythmic gene-expression programs.

## Figures and Tables

**Table 1:** qPCR primers.

Primer	Sequence	Primer	Sequence
M-Bmal1-F	CTTGCAAGCACCTTCCTTCC	M-Bmal1-R	GGGTCATCTTTGTCTGTGTC
M-Per1-F	TGAAGCAAGACCGGGAGAG	M-Per1-R	CACACACGCCATCACATCAA
M-Per2-F	GCGAAGCGCTTATTCCAGAG	M-Per2-R	AGTCTGAAGGCATCATCAGG
M-Actin-F	GGCTGTATTCCCCTCCATCG	M-Actin-R	CCAGTTGGTAACAATGCCATGT
M-Cry1-F	CACTGGTTCCGAAAGGGACTC	M-Cry1-R	CTGAAGCAAAAATCGCCACCT
M-Cry2-F	CACTGGTTCCGCAAAGGACTA	M-Cry2-R	CCACGGGTCGAGGATGTAGA
M-Prkca-F	GAACACATGATGGACGGGGT	M-Prkca-R	TCCTTTGCCGCACACTTTGG
M-Egfr-F	CCTGGTATGGGTATGAAAGA	M-Egfr-R	TGGTCATCCTCCTGTGAG
M-Mmp2-F	ATAACCTGGATGCCGTCGT	M-Mmp2-R	AGGCACCCTTGAAGAAGTAGC
M-Creb3-F	AGCCTTGTGCTTCGTTCTGGTG	M-Creb3-R	CATCGTAGAACAGTAGGCTTCGG
M-Col1a1-F	CCTCAGGGTATTGCTGGACAAC	M-Col1a1-R	CAGAAGGACCTTGTTTGCCAGG
M-Col1a2-F	GGCCCTCAAGGTTTCCAAGG	M-Col1a2-R	CACCCTGTGGTCCAACAACTC
M-Col3a1-F	GACCAAAAGGTGATGCTGGACAG	M-Col3a1-R	CAAGACCTCGTGCTCCAGTTAG

## Data Availability

The original processed data supporting the figures and conclusions of this study are included within the article. Raw RNA-seq count matrices and unprocessed sequencing files have not been deposited in public repositories such as GEO or SRA due to ongoing related follow-up analyses. All raw data used for figure generation and RNA-seq profiling are available from the corresponding author upon reasonable request.

## Abbreviations

The following abbreviations are used in this manuscript:

LD	Light/Dark
DD	Dark/Dark
BMAL1	Basic Helix-Loop-Helix ARNT Like 1
CLOCK	Circadian Locomotor Output Cycles Kaput Gene
Per1	Period1
Cry1	Cryptochrome1
Per2	Period2
Cry2	Cryptochrome2
TTFL	Transcription–Translation Feedback Loops
SCN	Suprachiasmatic Nucleus
ZT	Zeitgeber Time
KEGG	Kyoto Encyclopedia of Genes and Genomes
PCR	Polymerase Chain Reaction
Egfr	Epidermal Growth Factor Receptor
Prkca	Protein Kinase C Alpha
Mmp2	Matrix Metalloproteinase-2
Creb3	Cyclic Amp-Responsive Element-Binding Protein 3
Col1a1	Collagen Type I Alpha 1 Chain
Col1a2	Collagen Type I Alpha 2 Chain
Col3a1	Collagen Type III Alpha 1 Chain
